# Efficacy and safety of XEN®—Implantation vs. trabeculectomy: Data of a “real-world” setting

**DOI:** 10.1371/journal.pone.0231614

**Published:** 2020-04-20

**Authors:** Felix Mathias Wagner, Alexander Karl-Georg Schuster, Julia Emmerich, Panagiotis Chronopoulos, Esther Maria Hoffmann

**Affiliations:** Department of Ophthalmology, University Medical Center of the Johannes Gutenberg-University Mainz, Mainz, Germany; Universita degli Studi di Firenze, ITALY

## Abstract

**Objective:**

To compare surgical success, postoperative intraocular pressure and complication rates between trabeculectomy and XEN gelstent surgery in a cohort of glaucoma patients in a typical clinical setting.

**Methods:**

A retrospective cohort study of consecutive patients with refractory open-angle glaucoma including patients who underwent either stand-alone XEN gelstent insertion with Mitomycin C or trabeculectomy with Mitomycin C between 2016 and 2018 at the University Eye Hospital Mainz, Germany. Primary outcome measure was the proportion of surgical success 1 year after surgery. Patients with an IOP ≤18mmHg, an intraocular pressure reduction of >20% and in no need of revision surgery or topical medication were considered a complete surgical success. If topical therapy was necessary, they were considered a qualified success. Multivariable logistic regression analysis was carried out for the primary outcome including gender, age, preoperative intraocular pressure and number of medication classes used preoperatively as adjustment variables.

**Results:**

171 eyes of 144 patients were included, including 82 eyes of 58 patients in the XEN group and 89 eyes of 86 patients in the trabeculectomy group. The primary outcome defined as the proportion of surgical success after 1 year (mean 11.1 months ± 2.2) was similar for both groups. The complete success proportion was 65.5% (95%-CI: 55.6–75.9%) in the trabeculectomy group, and 58.5% (95%-CI: 47.6–69.4%) in the XEN group and not statistically different in our analysis model (crude OR = 0.61; 95%-CI: 0.31–1.22; adjusted OR = 0.66; 95%-CI: 0.32–1.37). The intraocular pressure reduction, as secondary outcome measure, was higher in the trabeculectomy group (10.5 mmHg) compared to the XEN group (7.2 mmHg; p = 0.003) at the 12-month follow-up.

**Conclusion:**

Both XEN gelstent implantation and trabeculectomy show similar proportions of surgical success and of complications and are therefore both recommendable for clinical routine. However, trabeculectomy seems to be more effective in lowering intraocular pressure than the XEN implantation. A prospective randomized clinical trial is necessary to evaluate differences in the long-term clinical outcome.

## Introduction

Glaucoma is a major cause for visual impairment and blindness in industrialized countries, with an increasing number of people affected [1]. Initial treatment is usually conservative, aiming to reduce intraocular pressure (IOP) by application of topical medication or laser trabeculoplasty [2]. In case of patients with insufficient response to pharmacological therapy, several surgical procedures have been developed over the last decades to lower IOP. At present, trabeculectomy is the most frequently performed procedure [3], relieving the intraocular pressure by creating a scleral ostomy to the anterior chamber, thus enabling drainage to the subconjunctival space. Due to its effective reduction of the intraocular pressure and its cost efficiency, it is considered the reference standard in surgical treatment of glaucoma [4].

However, recent developments have led to an expansion of the therapeutic options. For instance, a new group of procedures is pursuing a less invasive approach, aiming to reduce possible complications. Minimal Invasive Glaucoma Surgery (MIGS) includes a variety of interventions, extending from miniaturized versions of trabeculectomy to minimally invasive shunt or bypass operations, differing from traditional tube shunt procedures through limited surgical manipulation of the sclera and the conjunctiva [5].

Moreover, another more invasive approach was introduced in the last years: the implantation of a tube shunt into the anterior chamber angle with drainage under the conjunctiva, i.e. using the XEN45® gelstent (Allergan, Dublin, Ireland) [6], a 6mm porcine gelatin implant with a 45 μm lumen. The stent is implanted ab interno and creates a drainage fistula to the subconjunctival space [7].

Few studies have yet compared both filtering surgeries, XEN gelstent implantation and trabeculectomy. To this date, no prospective randomized clinical trial exists that compares the two procedures [8, 9]. Most recently, Schlenker et al. found that there were no detectable evidence of differences in the risk of failure or safety profiles between the two surgical procedures [9]. However, due to the study’s multicentric retrospective approach, differences in post-operative management and surgical techniques cannot be ruled out.

The aim of this study was the comparison of surgical success, postoperative IOP development and complication rates between trabeculectomy and XEN gelstent surgery in a cohort of glaucoma patients at a University Eye Clinic in Germany.

## Methods

This is a retrospective cohort study of consecutive patients with refractory open-angle glaucoma (primary open-angle glaucoma, pseudoexfoliative pigment dispersion, or normal-tension glaucoma) who underwent either stand-alone gelstent insertion (XEN) (XEN45® gelstent Allergan, Dublin, Ireland) with Mitomycin C (MMC) or trabeculectomy (TE) with MMC between January 11, 2016, and February 22, 2018, by 2 experienced surgeons (EMH, PC) at the University Eye Hospital Mainz, Germany. Both surgeons are glaucoma specialists with long experience in glaucoma surgery including filtering surgery and angle surgery. Furthermore, they had performed XEN implantations for over 1 year prior to the study. Patients with XEN-implantation or with trabeculectomy were chronologically identified by an electronic surgical case register, and then confirmed by manual chart review. All data were fully pseudonymized before they were accessed. According to regional laws, the requirement for informed consent was waived by the ethics committee of the medical board of Rhineland-Palatinate.

Preoperative baseline characteristics were collected from patients’ files, corresponding physicians’ letters, and surgical reports. Collected characteristics included demographics and ocular characteristics (IOP used for decision for surgery [pre-operative IOP], number of different glaucoma medications, glaucoma diagnosis, history of previous cataract surgery). Follow-up data was obtained through chart review and correspondence with ophthalmologists engaged in patient follow-up.

### Inclusion and exclusion criteria

Patients above the age of 18 with primary open-angle glaucoma, pseudoexfoliation, pigment dispersion and normal-tension glaucoma were included. Patients who did not meet these criteria or had prior filtering glaucoma surgery were excluded.

### Ab interno gelatine microstent implantation

After disinfection with povidone iodine, 0.02 mg MMC (0.1 ml) was injected under the conjunctiva posterior to the area of the planned gelstent injection site (at least 9 mm from the limbus).

The fluid was then massaged further posterior to avoid contact with the vulnerable limbus. A main and a side-port paracentesis were made, and the anterior chamber was filled with viscoelastic (Healon® or Healon GV^®^). The injector was inserted through the main incision and the needle guided to the opposite side of the anterior chamber. The correct positioning of the entry side was verified gonioscopically and the surgeon aimed to punctate the sclera above the trabecular meshwork. The needle was then advanced through the sclera, emerging below the conjunctiva. The injector was rotated 90°, and then withdrawn from its implantation area without any shift movement during the maneuver. The correct placement of the gelstent in the anterior chamber was confirmed by a second gonioscopy. By moving the conjunctiva with curved blunt forceps, the mobility of the gelstent was tested and checked for its straight, free and mobile position under the tenon. The viscoelastic was removed from the anterior chamber, the paracenteses were hydrated, the anterior chamber was deepened, and the presence of a bleb was confirmed.

### Trabeculectomy

A fornix-based flap of the conjunctiva was dissected, a shallow groove was created directly behind the former conjunctival insertion, and 0.02 mg of MMC were placed posteriorly under the conjunctiva for 3 minutes using a 7 x 7 mm soaked sponge. A 4 x 4 mm scleral flap of partial thickness was prepared, and a temporal paracentesis was made. A sclerostomy was created and a peripheral iridectomy was performed. The scleral flap was closed with four 10–0 nylon sutures, two edge sutures and two side sutures stitched tangentially through the scleral flap and the adjacent sclera to allow aqueous humor to flow posteriorly [10]. The conjunctiva was closed with improved sutures in a meander-like fashion for fornix-based conjunctival flaps as described by Pfeiffer and Grehn [11]. The presence of a bleb was confirmed.

### Perioperative management

According to the Mainz protocol, all patients were instructed to stop the use of antiglaucomatous eye drops on the treated eye 2–4 weeks preoperatively. In order to reduce conjunctival inflammation, patients were advised to use unpreserved topical steroids 5 days 4 times daily preoperatively. In case of an IOP increase, patients and treating ophthalmologists were instructed to treat IOP spikes with oral acetacolamide. Patients were hospitalized for surgery and were seen daily in the postoperative course. The postoperative topical regimen was the same for both interventions: topical antibiotic prophylaxis for 1 week and prednisolone unpreserved eye drops 6 times daily, tapering off over a period of 3–6 weeks. Subconjunctival 5 FU injections were given at the discretion of the treating surgeon. Any necessary interventions (including laser suture lysis, and digital ocular compression posterior to the scleral flap increasing the scleral outflow) were performed on site during the inpatient stay.

### Outcome measures

The primary outcome was the proportion of surgical success at 1 year after XEN-implantation compared to trabeculectomy; we distinguished complete success and qualified success. The procedure was considered as failure if one of the following criteria was met: IOP >18mmHg, IOP reduction of less than 20% compared to the pre-operative IOP, hypotony (IOP at 5mmHg or less), revision surgery or loss of light perception.

Revision surgery or complication was defined as additional surgery requiring a return to the operating room (such as needling procedures). Postoperative in-clinic maneuvers or interventions, including laser suture lyses, were not considered failures. The use of IOP lowering medication was allowed.

Patients who did not fail these criteria and did not necessitate glaucoma medication post-operatively were considered as complete success. If post-operative pharmaceutical treatment was necessary to achieve adequate IOP lowering (IOP ≤18mmHg and IOP reduction more than 20%) but no surgery was necessary in the meanwhile, these cases were considered a qualified success.

### Statistical analysis

Subjects’ demographic and ocular characteristics, including age, sex, intraocular pressure, intraocular pressure lowering, complications, medication, pseudophakia and disease type, were described by mean, standard deviation, median and interquartile range for continuous variables and by absolute and relative frequencies for categorical variables.

Comparisons with respect to intraocular pressure and visual acuity between the two treatment groups (XEN vs. trabeculectomy) were performed with a clustered Wilcoxon-Test for independent samples using the Rosner-Glynn-Lee method [12]. This was conducted to adjust for the fact that both eyes of one patient could be included into the statistics. A clustered chi-square test was used for categorial variables. Confidence intervals for categorical variables were computed. Multivariable logistic regression analysis with generalized estimating equations was applied to evaluate associated factors with complete success including age, sex, operation method, preoperative IOP, medication classes preoperative and pseudophakia as independent variables. The data was clustered by patients to account for the fact that patients received either surgical treatment in one or in both eyes. The same was conducted for qualified success as outcome.

This is an explorative study and a p value of 0.05 or less was considered as statistically significant. Statistical analyses were carried out with R (version 3.5.2, Eggshell Igloo and the packages clusrank, clust.bin.pair) [13–15] and with SPSS (IBM Corp. Released 2016. IBM SPSS Statistics for Windows, Version 24.0. Armonk, NY: IBM Corp.).

## Results

A total of 171 eyes of 144 patients were included and underwent surgery between January 11, 2016, and February 22, 2018, including 82 eyes of 58 patients in the XEN group and 89 eyes of 86 patients in the trabeculectomy group.

No patient received a XEN-implantation in one and a trabeculectomy in the other eye. Two patients from the trabeculectomy group were lost to follow-up between the six month and the 1-year visit. The baseline characteristics and glaucoma characteristics were similar between the 2 patient groups but differed in age and the used number of medication classes (Table 1). The study population consisted of 103 women (60.2%) and 68 men (39.8%) between the age of 45 and 89 years. The mean age was 68.7 years. On average, patients with XEN implantation were 5.8 years older than those undergoing trabeculectomy. Preoperatively, the trabeculectomy group used 3 classes of medication (median). In the XEN group, 2 classes (median) were used. Patients scheduled for surgery had similar visual field defects. Mean deviation (MD) was 10.4±6.5dB in XEN group vs. 10.9±6.1dB in the TE group (p = 0.12). We were unable to detect evidence of differences for any other characteristics.

**Table 1:** Baseline characteristics.

Characteristic	Total (n = 171)	XEN (n = 82)	Trabeculectomy (n = 89)	p value*
Demographic				
Age, Median (IQR), yrs	71 (62.0–77.0)	73.0 (65.8–80.0)	67.2 (59.2–74.8)	0.002
Female sex % (no.)	60.2 (103)	63.4 (52)	57.3 (51)	0.51^x^
Preop. IOP Median (IQR), mmHg^y^	20.0 (17.0–25.0)	19.0 (16.8–25.0)	21.0 (17.0–27.0)	0.07
Medication classes, median (IQR)^z^	3.0 (1.0–4.0)	2.0 (1.0–3.0)	3.0 (2.0–4.0)	0.004^x^
Glaucoma type % (no.) and severity				0.38^x^
Primary open-angle	73.1 (125)	74.4 (61)	71.9 (64)	
Pseudoexfoliation	11.1 (19)	8.5 (7)	13.5 (12)	
Pigment dispersion	5.3 (9)	4.9 (4)	5.6 (5)	
Uveitic	1.2 (2)	0.0 (0)	2.2 (2)	
Normal tension	9.4 (16)	12.2 (10)	6.7 (6)	
Visual field (MD in dB (SD))	10.4 (6.5)	9.9 (6.8)	10.9 (6.1)	0.12
Pseudophakia (yes)	48.5 (83)	59.8 (49)	38.2 (34)	0.14^x^

IOP = intraocular pressure; IQR = interquartile range; SD = standard deviation

*If not stated otherwise: clustered Wilcoxon-Test

^x^Chi-square test

^y^At which the decision was made to proceed with surgery.

^z^Number of medication classes to lower IOP when indication for surgery was made.

### Primary outcome: Surgical success

After 1-year (mean 11.1 months ± 2.2) follow-up, complete success was 65.5% [95%-CI: 55.6–75.9%] in the trabeculectomy group, and 58.5% [95%-CI: 47.6–69.4%] in the XEN group (crude OR = 0.61; [95%-CI: 0.31–1.22], p = 0.16; adjusted OR = 0.66; [95%-CI: 0.32–1.37], p = 0.26), showing no evidence for a difference between the two groups for the primary outcome.

We couldn’t find any association between complete success at 1 year and gender (p = 0.62), age (p = 0.30), preoperative IOP (p = 0.44) or the number of medication classes used preoperatively (p = 0.81) in the multivariable statistical model. When evaluating the surgical procedures trabeculectomy and XEN separately in a statistical model, there was also no association of qualified success and gender (TE: p = 0.92; XEN: p = 0.45), age (TE: p = 0.26; XEN: p = 0.57), preoperative IOP (TE: p = 0.59; XEN: p = 0.50) or the number of medication classes used preoperatively (TE: p = 0.22; XEN: p = 0.72).

After 6 months (mean 5.3±1.5 months), the complete success proportion was descriptively higher in the trabeculectomy group (72.4% [95%-CI: 62.8–81.3%]) than in the XEN group (59.8% [95%-CI: 49.3–69.3%]) (crude OR = 0.50 [95%-CI: 0.25–1.002], p = 0.051; adjusted OR = 0.48 [95%-CI: 0.22–1.07], p = 0.07).

Similarly, the proportion of qualified success after 6 months was descriptively higher in the trabeculectomy group (81.6% [95%-CI: 72.9–89.2%]) compared to the XEN group (70.7% [95%-CI: 61.0–80.0%]) (crude OR = 0.51 [95%-CI: 0.24–1.10], p = 0.08; adjusted OR = 0.44 [95%-CI: 0.18–1.09], p = 0.08]). One year postoperatively, 72.4% (95%-CI: 62.7–81.8%) of the patients from the trabeculectomy group met the criteria for qualified success, in the XEN group, the proportion was 72.0% (95%-CI: 61.7–81.0%) with no evidence of difference between the two groups (crude OR = 0.81 [95%-CI: 0.39–1.69], p = 0.57; adjusted OR = 0.72 [95%-CI: 0.32–1.62], p = 0.43).

**Fig 1:**
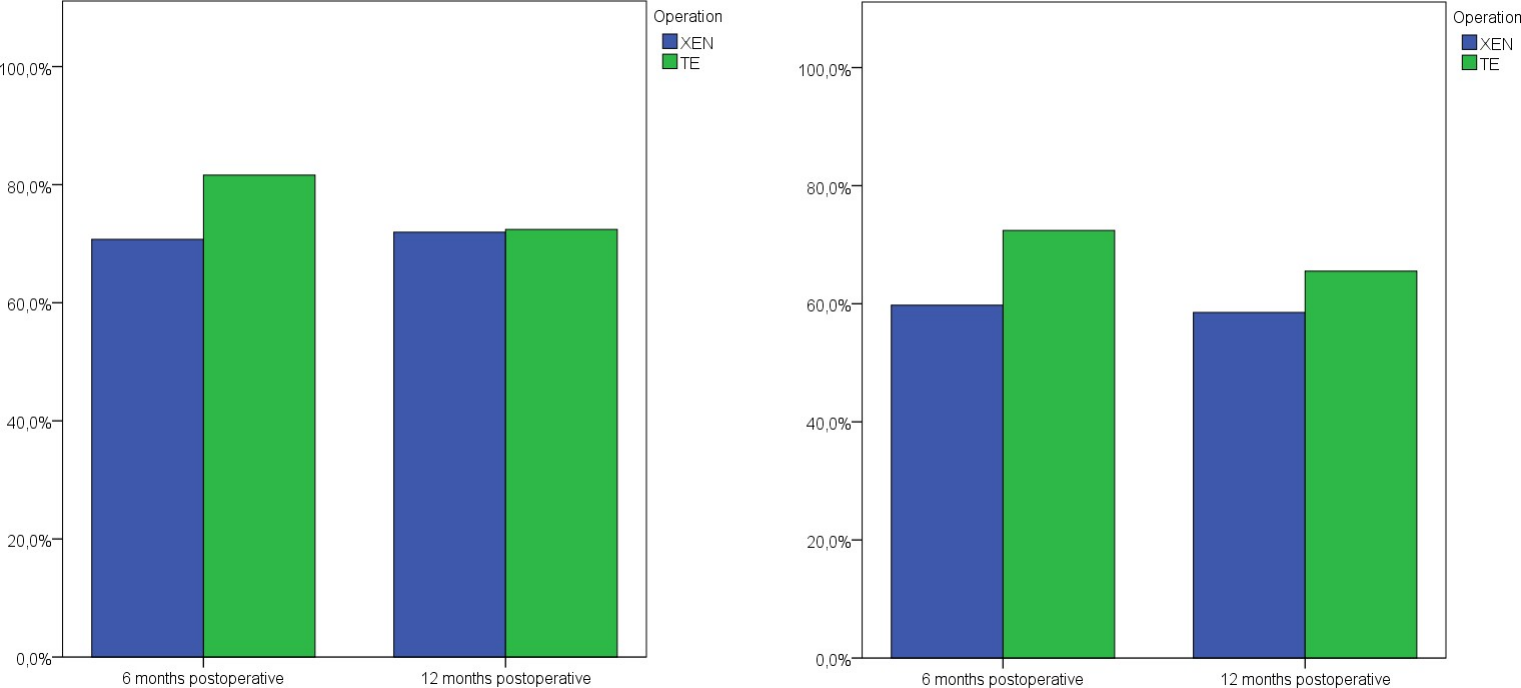
Surgical results: a) Proportion of qualified success, b) Proportion of complete success.

Success, qualified success and failure proportions for the two groups are shown in Fig 1. Reasons for surgical failure after 6 months and 12 months are presented in Table 2.

**Table 2:** Reasons for surgical failure after 12 months (% of failure).

	XEN (n = 23)	Trabeculectomy (n = 24)
Reoperation for IOP reduction	13 (56.5)	14 (58.3)
Inadequate IOP reduction without operation*	8 (34.8)	4 (16.7)
Hypotony	2 (8.7) ^†^	6 (25.0) ^‡^
Loss of light perception	0	0

*: with the use of medication

†: 1 of these patients received surgery for IOP increase

‡: 3 of these patients received surgery for IOP increase

### Secondary outcomes

**Fig 2:**
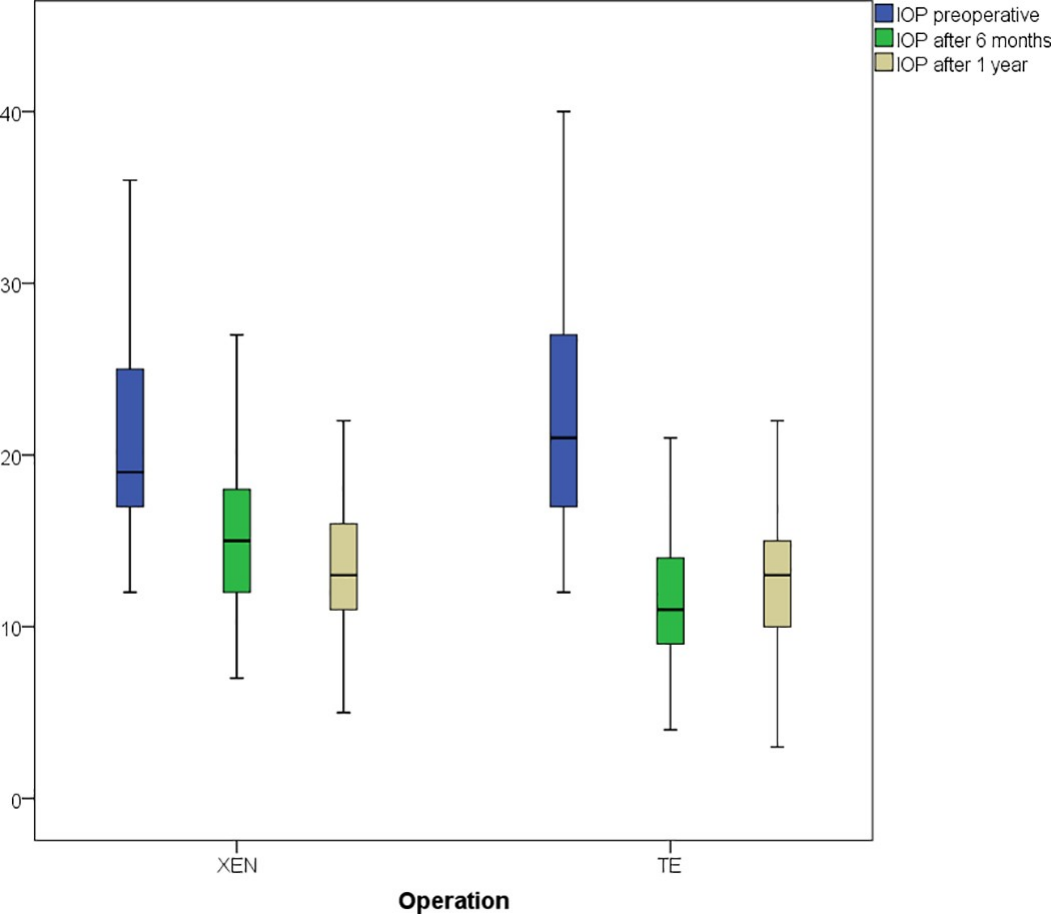
IOP development: IOP values (in mmHg), preoperatively (blue), after 6 months (green), after 1 year (ocher).

After 6 months, the IOP of the XEN participants was reduced by 5.5 ± 7.6 mmHg and by 11.9 ± 9.0 in the trabeculectomy group. The IOP reduction was significantly higher in the trabeculectomy group at the 6-month (p<0.001) and at the 12-month follow-up (p = 0.003). There, the IOP-reduction was 10.5 ± 9.2 mmHg in the trabeculectomy group and 7.2 ± 8.2 mmHg in the XEN group. IOP values for both groups are shown in Fig 2. After one year the XEN group used 0.3 ± 0.5 classes of medication, vs. 0.2 ± 0.4 classes of medication in the trabeculectomy group used (p = 0.17).

## Discussion

This retrospective cohort study of consecutive patients with refractory open-angle glaucoma conducted in a clinical setting compared the surgical success of trabeculectomy combined with MMC to the implantation of a XEN gelstent. We did not find evidence for a difference of complete surgical success between the two groups at 6 months and at 12 months, although the proportion of complete success was descriptively higher in the trabeculectomy group after 6 months (p = 0.06). With respect to qualified surgical success, we found similar results for the proportions after 6 months and one year.

Until today, few studies exist comparing the surgical outcome of trabeculectomy combined with MMC and the implantation of a XEN gelstent [8, 9, 16, 17]. Schlenker et al. compared the rate of surgical failure after trabeculectomy and XEN gelstent implantation, which directly corresponds to surgical success. The applied criteria for surgical failure by Schlenker et al. were similar to those reported in this study. Likewise, they found no statistical significant difference for surgical failure between the two groups [9].

The most common reason for surgical failure was the necessity for further surgery to lower IOP in both the trabeculectomy and XEN group. In most cases, needling or open XEN-revision was performed. The total rate of needling within one year after surgery was 16% in both groups. Similarly, Gedde et al. described a needling rate of 14% for their trabeculectomy cohort [18]. In contrast, Mansouri et al. reported a considerably higher needling rate of 45% in the first year after XEN-implantation [19]. One explanation for this difference could be the fact that we performed needling maneuvers as regular surgery in our operation facilities, whereas Mansouri et al. performed needling procedures at the slit lamp. Therefore, the barrier to perform needling might have been higher in our clinical setting.

There was no evidence for difference in the preoperative IOP between the two intervention groups. In both groups, the IOP was significantly reduced 6 months and one year after surgery. At both time points, patients having received trabeculectomy had a significantly higher IOP reduction compared to patients after XEN gelstent implantation. The changes in IOP after both procedures are similar to reported IOP changes in current literature [20–24]. Gedde et al. reported a mean IOP reduction of 12.9 mmHg one year after trabeculectomy, which is slightly higher than the reduction 10.5 mmHg we observed but within the standard deviation range [23]. Karimi et al. found a mean IOP reduction of 5.0 mmHg one year after XEN stent implantation, which is slightly lower than our reduction of 7.2 mmHg, but again within our standard deviation range [24]. It is therefore justified to state that both interventions show beneficiary outcomes.

On average, participants in the XEN gelstent group were 5.8 years older and used one medication classes less preoperatively. These differences may influence the results, as recently shown by Hoang et al. regarding the influence of age on filtrating surgeries [25]. However, the clustered logistic regression mixed model, adjusting for gender, age, preoperative IOD or the number of medication classes used preoperatively, did not alter our finding that there is no evidence of a difference in the proportion of success between both study groups.

Nonetheless, our study has several limitations. First, it is a single center retrospective study. Due to its retrospective nature, the lack of randomization can lead to a selection bias. However, patients in both groups showed comparable glaucoma damage, based on visual field exam. Even if the number of patients is reasonable high for a single center study, the sample size is still small considering the small differences between the two investigated methods. Conducted with a larger sample size, a comparison of trabeculectomy and XEN-implantation may reveal significant results for the indicated differences in our study. Moreover, the follow-up time is too low to assess sustained success. Therefore, long-term investigations are desired.

In conclusion, both XEN gelstent implantation and trabeculectomy in patients with refractory open-angle glaucoma show comparable surgical success proportions and similar proportions of complications and are therefore both recommendable for clinical routine. The reduction of IOP is higher for trabeculectomy than for XEN gelstent. A prospective randomized clinical trial, similar to the clinical trial on “InnFocus microshunt versus trabeculectomy study” [26] is needed to investigate whether filtering glaucoma surgery or XEN gelstent implantation are superior regarding complete and qualified success rates in glaucoma treatment. Until then, the decision should be made individually and together with an informed patient.

## Supporting information

S1 Data (SAV)
